# MAIN CONTROVERSIES IN THE NONOPERATIVE MANAGEMENT OF BLUNT SPLENIC INJURIES

**DOI:** 10.1590/0102-6720201600010016

**Published:** 2016

**Authors:** Jorge Roberto Marcante CARLOTTO, Gaspar de Jesus LOPES-FILHO, Ramiro COLLEONI-NETO

**Affiliations:** Surgical Gastroenterology Division, Department of Surgery, School of Medicine, Federal University of São Paulo (EPM/UNIFESP), São Paulo, SP, Brazil

**Keywords:** Spleen, Therapeutics, Blunt trauma, Abdomen, Hemorrhage

## Abstract

***Introduction*:**

The nonoperative management of traumatic spleen injuries is the modality of choice in patients with blunt abdominal trauma and hemodynamic stability. However, there are still questions about the treatment indication in some groups of patients, as well as its follow-up.

**Aim:**

Update knowledge about the spleen injury.

***Method*:**

Was performed review of the literature on the nonoperative management of blunt injuries of the spleen in databases: Cochrane Library, Medline and SciELO. Were evaluated articles in English and Portuguese, between 1955 and 2014, using the headings "splenic injury, nonoperative management and blunt abdominal trauma".

***Results*:**

Were selected 35 articles. Most of them were recommendation grade B and C.

***Conclusion*:**

The spleen traumatic injuries are frequent and its nonoperative management is a worldwide trend. The available literature does not explain all aspects on treatment. The authors developed a systematization of care based on the best available scientific evidence to better treat this condition.

## Abstract (pt)

***Introdução*:**

O tratamento não operatório das lesões traumáticas do baço é a modalidade de escolha nos pacientes com trauma abdominal contuso e estabilidade hemodinâmica. No entanto, ainda existem dúvidas sobre a indicação do tratamento em determinado grupo de pacientes, assim como o seu seguimento.

**Objetivo:**

Atualizar o conhecimento sobre as lesões do baço.

***Método*:**

Realizou-se revisão da literatura sobre o tratamento não operatório das lesões contusas do baço nas bases de dados: Cochrane, Medline e SciELO. Foram incluídos os artigos em línguas portuguesa e inglesa entre 1955 e 2014, utilizando os descritores "splenic injury, nonoperative management e blunt abdominal trauma".

***Resultado*:**

Foram selecionados 35 artigos. A maioria dos trabalhos eran em grau de recomendação B e C.

***Conclusão*:**

As lesões traumáticas do baço são frequentes e o seu tratamento não operatório apresenta tendência mundial. A literatura disponível não esclarece todos os aspectos deste tratamento. Elaborou-se sistematização de atendimento baseada nas melhores evidências científicas disponíveis para facilitar seu manejo.

## INTRODUCTION

The spleen is the most injured body organ when there is a direct impact on the left upper quadrant, leading to intense intraperitoneal hemorrhage and shock, even though its location is well protected by costal grid^16^. The treatment can be operative and non-operative.

For many years, the main focus was the control of bleeding and splenectomy was the performed regardless the type of injury. In the 1980s occurred continuous surgeon efforts trying to preserve the splenic tissue in trauma victims, based on studies that demonstrated the importance of the spleen in the immune and hematopoietic system and motivating conservative operations, such as splenorraphy and segmental resection. From the 1990s, several factors contributed to the success of non-operative treatment (TNO) of these injuries as the best hospital conditions, the spread of initial care, life support to multiple trauma, improvement of computed tomography and angioembolization technique^12^.

The TNO of traumatic spleen injuries is the gold standard method in patients with blunt abdominal trauma and hemodynamic stability^16^^,^ 19 ^,^ 33. Nevertheless, there are still doubts in some groups of patients, as well as inpatient and outpatient follow-up.

The objective of this review is to update the knowledge of this entity, of great interest to the present lifestyle.

## METHOD

Literature review was performed in Cochrane, Medline and SciELO. The Cochrane Database was screened by the Virtual Health Library (cochrane.bireme.br). The Medline by National Library of Medicine and National Institutes of Health using Entrez PubMed (www.pubmed.gov). The SciELO accessed by Scientific Electronic Library Online (www.scielo.org). The initial search identified articles in English and Portuguese, between 1955 and 2015, using the keywords "splenic injury, non-operative manegement and abdominal blunt trauma". Were selected 35 articles for the review of the major TNO controversies on spleen traumatic injurie

Studies were ranked by degree of recommendation (Table 1) of the Oxford Centre for Evidence-based Medicine Levels of Evidence (2009)^22^.

**TABLE 1:** - Recommendation degrees of selected articles on TNO of traumatic injuries of the spleen

**Recommendation degrees**	**Number of articles (%)**
A	None (0)
B	10 (28.57)
C	19 (54.28)
D	6 (17.14)

No randomized clinical trial on the topic was localized. Among the articles selected there are two meta-analyzes, two systematic reviews, 25 observational studies, four population surveys and two guidelines. Among the observational studies all were retrospective, six were multicentre and 84% had more than 100 patients. In observational studies, 24% were case-control.

### TNO on traumatic lesions of the spleen

TNO has increased over the past 15 years. In 1997, Peitzman et al. reported that 54.8% of patients were treated with non-surgical form; Smith et al. indicated TNO up to 80%^23^^,^ 32.

This treatment modality is associated with lower hospital costs, fewer non-therapeutic laparotomy, a lower rate of intra-abdominal complications, lower rates of blood transfusion and decreased morbidity and mortality^10^^,^ 12. TNO is only recommended if the institution is able to follow the patient continuously with various examinations done by experient medical supervision^10^^,^ 16.

There are still some controversies in the issue. The main ones are:

### Grade of splenic injury 

TNO have been of choice in hemodynamically stable patient, regardless of the degree of injury^16^^,^ 19 ^,^ 33, although there is a direct correlation between the degree of splenic injury and the percentage of failure. Peitzman et al. demonstrated, through multicenter study of 1488 patients, success in 75% in grade I lesion; 70% with grade II; 49.3% with grade III; 16.9% with grade IV; and 1.3% with grade V^23^. In another study, 224 patients with lesions grade IV and V were subjected to 62% success with TNO^35^. Fernandes et al. conducted TNO in 94 patients with grade IV, with good results in 92.3% with strict protocol application^10^. Rosati et al., in eight years of experience, TNO was performed in 67.6% of multiple trauma with injuries grade IV or V^28^. Hsieh et al. were successful in 39 of 42 patients with lesions grade III, IV and V^11^. The guideline of The Eastern Association for the Surgery of Trauma (EAST) does not contraindicate the TNO in patients with severe spleen injury on CT and stable hemodynamically^33^. 

### Hemoperitoneum volume 

The volume of hemoperitoneum in patients with stable hemodynamic parameters is not considered a contraindication to TNO, according to EAST^33^. The articles Peitzman et al. and Bhangu et al. reported that the hemoperitoneum volume can be predictive of failure, but also do not recommend it as a criterion for contraindication^3^^,^ 23. 

### Contrast extravasation on CT 

This overflow with splenic injury does not indicate requirement of surgical approach, since the patient is hemodynamically stable. EAST noted that the contrast extravasation alone is not an absolute indication for surgery or arteriography, and that other factors must be taken into account, such as age, the degree of injury and the presence of arterial hypotension^33^. Post et al. demonstrated that in grade I or II lesions, contrast extravasation on CT was not associated with worse results^25^. Peitzman et al. reported that 85% of patients with contrast extravasation on CT required surgical intervention, either on admission or in follow-up^23^. In the study among EAST experts, 82.6% of them carry out interventionist manouver (surgery or arteriography) in patients with contrast extravasation on inicial CT^9^. Patients with intraperitoneal extravasation have a higher chance of hemodynamic instability^11^. It is a specific marker for active bleeding and can predict the need for early intervention^5^. Therefore, the contrast extravasation on CT is an important sign of TNO failure probability, but should not be evaluated alone.

### Upper age limit

Age is no single criterion for TNO. Previously, the elderly were excluded from the recommendations because of the high failure rates obtained by authors in patients over 55 years^1^. The ideal posture of TNO in the elderly should be rigid because of the difficulty of estimating the specific spleen weakness and diminished physiological reserve in this population. Bhullar et al., studying 80 patients over 55 years, reported that age was not an independent risk factor for TNO failure^4^. Fernandes et al. demonstrated success rate of 83.33% for grade IV lesions in patients over 55 years^10^. EAST considers that the age of 55 years is not contraindication^33^. Olthof et al., analyzing questionnaire given to 30 experts in trauma surgery or interventional radiology, found that age did not influence the therapeutic strategy^21^. The Gomez et al. questionnaire, directed to 70 experts in the treatment of splenic trauma, found that for 97% the age is not considered a contraindication^12^.

### Injury Severity Score (ISS)

In hemodynamically stable patients, although not contraindicated, high ISS has a higher chance of failure in TNO. Studies have shown that patients with ISS greater than 15 are more likely to require operation and have TNO failure^23^^,^ 24 ^,^ 36. Contrary to these findings, some experts believe that ISS does not influence the therapeutic strategy of splenic injuries^21^.

### Severe traumatic brain injury

Another aspect controversial in TNO indication refers to patients with severe traumatic brain injury. Shapiro et al. demonstrated that TNO can be done successfully in hemodynamically stable patients with neurological injury, where the level of awareness did not represent formal contraindication to this handling^31^. In report done by Fernandes et al., including patients with splenic injury grade IV, some patients had severe head injury and there has been no failure in TNO^10^. Olthof et al. reported that there is consensus among experts about the non-interference of the level of consciousness in the splenic injury treatment decision^21^. Gomez et al. mentioned that 64% of specialists perform TNO in the presence of severe head trauma, but they emphasize that this decision is dependent on the ICU quality present in treatment^12^. The Western Trauma Association described the contraindications to the existing TNO in the past, such as neurological damage, were overcome^19^. EAST noted that the level of consciousness is not a contraindication^33^

### Number of transfused blood bags

There is a number of transfused blood bags which contraindicate TNO. It is an independent predictor of mortality in patients with polytrauma^27^. Peitzman et al. demonstrated in a multicenter study, patients who failed the TNO, received more blood bags during hospitalization than those who underwent treatment with success^23^. Gomez et al. mentioned that some experts agreed on the importance of the number of transfused blood units in the first 24 h, but not reached a consensus as to the number that contraindicate the method^12^. According Olthof et al., transfusion of five or more units of blood would be needed to influence the decision on the type of trauma treatment^21^. The EAST guideline considers that the number of blood bags against TNO is still a matter not answered^33^.

### Follow-up of patients in the spleen injury treated with TNO

The hospital component is critical to the realization of TNO. Follow-up with strict protocols are essential. According Peitzman et al., only a third of trauma centers have well established TNO protocol for spleen injuries^23^. After five years, only 29.9% of the experts on EAST do have it^9^. Only 20.4% of experts from The American Association for the Surgery of Trauma consider that the protocol used in their institutions is well supported by the literature^36^.

### Inpatient unit

Ideally, the patient selected for the TNO should stay in the ICU or in units with continuous monitoring. The institutional protocol presented by Fernandes et al., all patients should be admitted to the ICU^10^. The survey of Olthof et al. showed that 100% of the surveyed experts admit patients in units with continuous monitoring of vital signs and 63% of them in the ICU. The experts answered that the duration of admission is determined by the clinical situation and the TNO hospital protocol, but 96% agree to keep monitoring at least three days^21^. Gomez et al. reported that 78% of respondents experts admit the patient in ICU^12^. For EAST consulted members, 75% agree that patients with grade II injury spleen should be admitted to the ICU^9^. London et al. showed that protocols that incorporate long periods in bed are unnecessary, since the time of mobilization of patients in TNO did not contribute to late bleeding^15^. There are still doubts as to the time required for continuous monitoring of these patients^33^.

### Surgical team

Surgical team must be available 24 h a day; this is a basic requirement for TNO spleen trauma. Its success depends on clinical examination and cases, if possible, should be followed by the same team that received the patient^16^. The EAST guideline refers to the need of the physical serial examination by the surgical team, but there are doubts as to the timing^33^.

### Hematimetria

The EAST guideline defines the need for hematocrit monitoring during hospitalization, but remains uncertain in duration and frequency of this mensuration^33^. Gomez et al. reported that 85% of the experts who answered the questionnaire monitor hemoglobin every 8-12 h^12^. The measurement may be performed every 6 h on the first day, every 12 h until the third day and every 24 h until hospital discharge^16^. Olthof et al. showed that all the experts who participated in the survey perform serial measurement. In the first 24 h, the measurement is performed every 4-6 h. After the first 24 h, every 12-24 h^21^. Fernandes et al. used the monitoring every 6 h during the first 24 h or more often if there were signs of deterioration^10^.

### Diet

The return to the diet is critical in trauma patients. The guideline EAST states that the opening of the oral diet has still doubts in literature^33^. Gomez et al. mentioned that 71% of specialists initiates oral diet in stable patients clinically after 24 h of trauma^12^.

### DVT prophylaxis (deep vein thrombosis)

Patient with multiple trauma has increased risk of thromboembolic complications. Rostas et al. reported in a retrospective study of 328 patients, the early use of low molecular weight heparin was not associated with bleeding and TNO failure^29^. Another study suggested that the use of low molecular weight heparin in the first 48-72 h of admission was not associated with increased need for blood transfusion nor TNO failure^7^. Thus, only a minority does not use pharmacologic prophylaxis^36^. The guideline EAST states that despite some evidence that the chemical prophylaxis for DVT does not negatively interfere with TNO, there is no consensus in the literature about the safest time for its start after trauma^33^.

### CT control

CT control in successfully treated patients with non-operative form has no benefit. Haan et al. reported that it has no benefit in clinically stable patients with low splenic injuries^13^. There is no consensus among experts as to the realization of a new CT; 46% recommend new imaging, especially for the detection of vascular non-bleeding lesions^21^. Fata et al. showed that only 14.5% of surveyed surgeons performed control CT following the TNO^9^. It should be performed in patients with persistent systemic inflammatory response signals, persistent abdominal pain, suspected bowel injury, unexplained fall in hemoglobin and hematocrit levels or deterioration in the clinical status^10^^,^ 33. It can also be routinely performed if there was contrast extravasation at the first examination in the presence of subcapsular hematoma in the initial examination, underlying splenic disease, coagulopathies and athletes^33^.

### Hospital discharge

The time for discharge is also not well established in the literature. Fata et al. found that clinical judgment is the predominant^9^. A systematic review of Cirocchi et al. showed that the length of stay in the non-operative form of treated is less than with splenectomies^6^. In the survey conducted by Olthof et al., 100% of experts agree that the most important factors in determining the length of hospital stay are the stability of vital signs and hemoglobin^21^. McCray et al. reported through 449 patients, 96% success in TNO using as discharge criterion the hematologic stability and not the time after trauma^17^. The survey of Gomez et al. reported that 88% of the experts discharge patients prior to seven days inhospital^12^. The policy of EAST has not set the time required for hospitalization, it is subject that needs more studies^33^.

### Return to activities 

Barring the activities is recommended common in the spleen trauma victims after hospital discharge. Although most authors directly relates the duration of this period with the severity of splenic injury, there is no consensus in the literature on this point. Fata et al. reported that most experts use two weeks to the resumption of activity in patients with low-grade lesions and six in high-grade lesions. The biggest question would be in patients with lesions grade III, IV and V, where some adopt the three-month period. For these recommendations, particularly if used clinical judgment and rarely a picture control^9^. Gomez et al. showed that 67% contraindicate the return to activities before four weeks^12^. The investigation by Zarzaur et al. mentions that despite the consensus on the need to consider the type of activity performed by the patient, as well as the degree of injury to set the time off, the disagreement persists mainly in IV and V grade lesions. Some recommended permanent leave to sports^36^. In another study, they mentioned often recommend removal of three months, but this fact did not represent the majority^21^. Most protocols defines the clearance time according to the degree of injury. The guideline EAST dont set this aspect, highlighting the lack of consensus in the literature and suggests that this issue be the subject of investigations in the future^33^. 

### Arteriography with embolization of the splenic artery (AEAE) 

In recent decades, interest in splenic preservation increased and was facilitated by the improvement of the procedure and increasing the number of specialists who perform it. The implementation of new technologies such as AEAE increased spleen preservation rate after traumatic injuries and diminished TNO failure^3^^,^ 37. Using this method occurred reduction of splenectomy and was recognized as an independent predictor of splenic preservation in patients selected for TNO^2^^,^ 34. The arteriography with embolization is not free of complications, so its benefit in splenic trauma must be weighed against the hemodynamic deterioration during angiography, late control of hemorrhage, complications of the procedure, doubts regarding the preservation of splenic function after procedure, intra-abdominal injuries and unnoticed own failure rate of arteriography embolization^8^. AEAE indication consensus has not yet been established in the literature in spleen trauma. Even the absence of universally accepted algorithm, most centers indicate it in patients with contrast extravasation on CT, splenic injury grade IV or V and non-bleeding vascular lesions, such as pseudoaneurysm of the splenic artery and arteriovenous fistula 2 ^,^ 8 ^,^ 18 ^,^ 34 . The presence of large CT hemoperitoneum can also be an indication for it^2^. The guideline EAST indicates the procedure in patients with splenic injury with greater degree than III, contrast extravasation presence in CT, moderate hemoperitoneum, those patients with predictive factors of TNO failure and vascular lesions in non-bleedings^19^^,^ 33. Through opinions, Olthof et al. indicate AEAE in contrast extravasation and non-bleeding vascular lesions, but the most important condition for the indication would be available 24 h a day experienced staff in intervencionist radiology^21^. AEAE results depends on more comprehensive or selective indication, but there is a tendency for positive results in the splenic preservation. Requarth et al. conducted a meta-analysis with 10,157 patients from nine selected articles and concluded that AEAE was associated with high rates of splenic preservation in traumatic injuries grade IV and V^26^. Zarzaur et al. conducted a retrospective study of 10,405 patients in different centers of angiography and concluded that AEAE has a protective effect on the preservation of the spleen, especially the earlier it is realized^37^. Miller et al. prospectively studied 168 patients with splenic injury grade III to V and concluded that the routine use of AEAE in grade lesions III to V decreased the preservation of failure rates^18^. High success rates in the TNO of spleen traumatic injuries are also influenced by the selective use of AEAE^2^. 

### A proposal for systematizing the TNO on traumatic spleen injuries

**FIGURE 1:**
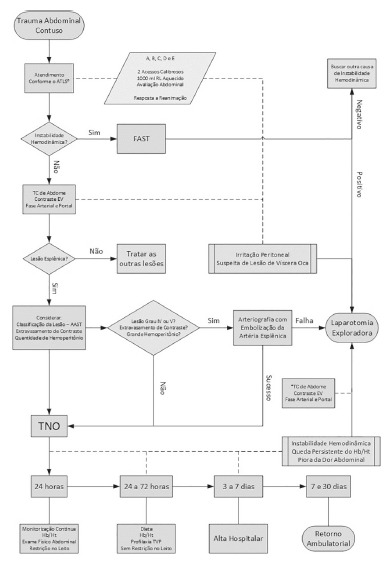
- Proposed TNO flowchart in traumatic injuries of the spleen

The authors of this paper have proposed to systematize the TNO after critical review of the literature for use in Brazilian hospitals. It consists of a patient care flow chart with blunt abdominal trauma (Figure 1), a hospital following model of these patients (Table 2) and recommendations regarding return to activities of patients who underwent the TNO (Table 3).

*In TNO failure, the patient may be referred for CT of the abdomen prior to laparotomy, since hemodynamically stable

**TABLE 2:** - Follow-up of patients with splenic injury treated with non-operative form

**Patients follow-up**			
Patient care	24 h	24 to 72 h	3 a 7 days
Continuous monitoring	Yes	6/6 h	Routine
Hb/Ht	6/6 h	12/12 h	Daily
Abdominal examination	4/4 h	6/6 h	12/12 h
Diet	Fasting	Oral or enteral	Oral or enteral
Pharmacological prophylaxis of DVT	No	HNF or HBPM	HNF or HBPM
Restraint in bed	Yes	No	No

UFH = unfractionated heparin; LMWH = low molecular weight heparina

**TABLE 3:** - Withdrawal activities time in relation to the degree of splenic injury in TNO of spleen traumatic injuries

**Type of activity**	**Lesion grade**	**Time to return**
Usual effort	I to V	2 weeks
Physical effort	I, II and III	2 months
	IV and V	3 months
Contact sports	I, II and III	6 months
	IV and V	12 months

## CONCLUSION

Spleen traumatic injuries are frequent and TNO has a worldwide trend. Although the available literature, some questions were unclear and there is a need to develop studiesneed with the best grade of recommendation. Thus, the authors developed care systematization based on the best available scientific evidence.
